# A recombinant lipid transfer protein from *Cannabis sativa*: IgE-binding properties in patients with symptoms to *Cannabis*

**DOI:** 10.1186/2045-7022-4-S2-P57

**Published:** 2014-03-17

**Authors:** Hans-Peter Rihs, Alicia Armentia Medina, Wolfram Lobin, Ingrid Sander, Thomas Brüning, Monika Raulf-Heimsoth, Rita Varga

**Affiliations:** 1 Institute for Prevention and Occupational Medicine of the German Social Accident Insurance (IPA), Molecular Genetics, Bochum, Germany; 2 Rio Hortega University Hospital, Allergy Unit, Valladolid, Spain; 3 Botanical Institute, University of Bonn, Botanical Garden, Bonn, Germany; 4 Institute for Prevention and Occupational Medicine of the German Social Accident Insurance (IPA), Allergology/Immunology, Bochum, Germany; 5 Institute for Prevention and Occupational Medicine of the German Social Accident Insurance (IPA), Head of the Institute, Bochum, Germany; 6 Ludwig-Maximilians-University (LMU), Clinics for Dermatology and Allergology, Munich, Germany

## Background

Due to the increasing social, medical and occupational exposure to *Cannabis sativa*, allergic reactions grow in frequency but little is known about the IgE-reactivity of single *Cannabis* allergens.

## Methods

To identify the mature peptide sequence of the lipid transfer protein (LTP) and to study the IgE-binding reactivity of a recombinant Can s 3 (rCan s 3), a cDNA was synthesized from total RNA of *Cannabis sativa L. ssp. sativa cv. Kompolti* leaves obtained from the botanical garden of the University Bonn. The LTP gene was amplified with a primer mix deduced from published amino acid sequences. The LTP variant was identified in five independent clones by sequencing in the pDrive vector system followed by the expression of the mature LTP in the pMAL-vector. After isolation of a soluble recombinant maltose-binding protein (MBP)-Can s 3 fusion protein, aliquots were biotinylated and coupled to Streptavidin-ImmunoCAPs.

## Results

Sera of 16 (6 Spanish and 10 German) subjects with allergic symptoms to *Cannabis* were tested. Specific IgE (sIgE) values >0.35 kUA/L were regarded as positive. Twelve out of 16 sera (75%) showed sIgE to *Cannabis* (range: 0.42-31.80 kUA/L). Five of them (31%) displayed sIgE to rCan s 3 (range: 0.40-14.10 kUA/L) but no sIgE to MBP. Specific IgE-reactivity to Pru p 3 was detected in all *Cannabis*-positive sera from Spain but only in 3 out of 6 German sera. All sera with sIgE to rCan s 3 showed also sIgE to Pru p 3.

## Conclusions

These results show for the first time IgE-binding of a recombinant *Cannabis* allergen (rCan s 3). Due to the 60% amino acid identity between Can s 3 and Pru p 3 a cross-reactivity is possible, but also to the LTPs of other plants. Since rCan s 3 is now available for sIgE diagnostics, implementation in larger studies may help to further elucidate the impact of this allergen.

